# A systematic review and meta-analysis of the prevalence of hepatitis C virus infection in people who inject drugs in Iran

**DOI:** 10.1186/s12889-020-8175-1

**Published:** 2020-01-14

**Authors:** Masoud Behzadifar, Meysam Behzadifar, Nicola Luigi Bragazzi

**Affiliations:** 1 Social Determinants of Health Research Center, Lorestan University of Medical Sciences, Khorramabad, Iran; 2 Health Management and Economics Research Center, Iran University of Medical Sciences, Tehran, Iran; 3 School of Public Health, Department of Health Sciences (DISSAL), University of Genoa, Genoa, Italy

**Keywords:** Hepatitis C virus, Meta-analysis, Harm reduction policies, Health management, Iran

## Abstract

**Background:**

Hepatitis C virus (HCV) infection is one of the major public health challenges generating a relevant burden. High-risk groups, including people who inject drugs (PWID), are at serious risk for developing HCV. In recent years, several investigations have been conducted in Iran to assess the prevalence e of HCV among PWID. The aim of the present study was to synthesize the literature performing a comprehensive search and meta-analysis.

**Methods:**

A comprehensive literature search was carried out from January 2000 to September 2019. Several international databases, namely Scopus, PubMed/MEDLINE, Embase, ISI/Web of Science, PsycINFO, CINAHL, the Cochrane Library and the Directory of Open Access Journals (DOAJ), as well as Iranian databases (Barakathns, SID and MagIran), were consulted. Eligible studies were identified according to the following PECOS (population, exposure, comparison/comparator, outcome and study type) criteria: i) population: Iranian population; ii) exposure: injection drug users; iii) comparison/comparator: type of substance injected and level of substance use, iv) outcome: HCV prevalence; and v) study type: cross-sectional study. After finding potentially related studies, authors extracted relevant data and information based on an ad hoc Excel spreadsheet. Extracted data included the surname of the first author, the study journal, the year of publication, the number of participants examined, the type of diagnostic test performed, the number of positive HCV patients, the number of participants stratified by gender, the reported prevalence, the duration of drug injection practice and the history of using a shared syringe.

**Results:**

Forty-two studies were included. 15,072 PWID were assessed for determining the prevalence of HCV. The overall prevalence of HCV among PWID in Iran was computed to be 47% (CI 95: 39–56). The prevalence ranged between 7 and 96%. Men and subjects using a common/shared syringe were 1.46 and 3.95 times more likely to be at risk, respectively.

**Conclusion:**

The findings of the present study showed that the prevalence of HCV among PWIDs in Iran is high. The support and implementation of ad hoc health-related policies and programs that reduce this should be put into action.

## Background

Health policy- and decision-makers consider hepatitis C virus (HCV) infection as one of the major health challenges in the field of public health, in that it generates a relevant burden, both in epidemiological and clinical terms [1]. High-risk groups such as prisoners, people with HIV, those who receive blood products and people who inject drugs (PWID) are at serious risk for HCV [2]. The World Health Organization (WHO) estimates that in 2015 around 1.75 million new cases of infections occurred worldwide. According to the WHO, the highest prevalence of infection was reported in the Eastern Mediterranean (2.3%), and in the European (1.5%) regions [3].

Among the high-risk groups for HCV, PWID represent a category that needs to be monitored and checked carefully [4]. Unsafe injection is one of the main ways of transmitting HCV infection [5], in particular, the usage of common syringes, which is quite a widespread practice among PWID [6]. The risk of HCV infection among these people is higher than the risk among HIV patients. Identifying high-risk groups can greatly help the healthcare system prevent and control various communicable diseases [7, 8].

Chronic HCV infection can cause cirrhosis, hepatocellular carcinoma and ultimately lead to death [9]. Due to the severe clinical outcomes, the high costs of the treatment and the absence of effective vaccines for HCV, health policy- and decision-makers tend to especially focus on prevention, control and management of HCV patients [10]. The WHO has identified the HCV elimination plan for 2030 as an important, ambitious goal, and as such, one of the most important ways to achieve this goal is to screen and control the disease in high-risk groups such as PWID [11].

The Middle East region is one of the areas worldwide in which HCV is highly prevalent. The risk of the transmission and spreading of HCV among PWID has increased in the last years as a result of the transit of drugs and addicted people through Afghanistan and neighboring countries [12]. Iran is one of the countries in the Eastern Mediterranean region, with about 186,000 HCV patients [13]. According to a recently published systematic review and meta-analysis, the prevalence of HCV in Iran is about 0.6% [14]. Despite the fact that this rate is lower compared to many neighboring countries in the area, the rate among high-risk groups such as PWID has considerably increased and this is a serious warning for Iranian health policy- and decision-makers [15]. Neighborhood with countries like Afghanistan is one of the major causes of this increase, and the Iranian government has been trying to mobilize all its resources to cope with this challenge [13].

In recent years, several investigations have been conducted in Iran to assess the prevalence of HCV among PWID, in order to provide planners with good evidence that can be used to implement appropriate health-related policies [16]. Like other countries in the world, also in Iran people who use personal or common syringes for intravenous injections are defined as PWID [17].

Understanding the epidemiologic status can provide a clearer and more appropriate framework for decision-and policy-makers in the health sector. They can use this information to develop their programs and plans in the different areas of HCV control and management. Health policy-and decision-makers, using available evidence, can effectively curb the costs generated by HCV in their country.

The aim of the present study was to investigate the prevalence of HCV among PWID by performing a comprehensive literature search and meta-analysis of published studies and to critically evaluate and appraise the policies that the health sector has been trying to implement in order to reduce the burden of HCV in Iran.

## Methods

### Systematic review and meta-analysis study protocol

The study protocol has been prospectively registered within the PROSPERO database (identification ID: CRD42019122601) [18] and the main findings are here reported in accordance with the “Preferred Reporting Items for Systematic Reviews and Meta-Analyses” (PRISMA) guidelines [19].

### Search strategy

A comprehensive literature search has been carried out in order to retrieve relevant studies related to the topic under study, from January 2000 to September 2019. Several international databases, repositories and bibliographic *thesauri*, namely Scopus, PubMed/MEDLINE, Embase, ISI/Web of Science, PsycINFO, CINAHL, the Cochrane Library and the Directory of Open Access Journals (DOAJ), as well as Iranian databases (Barakathns, SID and MagIran), have been mined independently by two researchers. To minimize the chance of not capturing all relevant studies and to find more potentially related studies, also the gray literature was consulted via Google Scholar. Furthermore, references lists of each potentially eligible study were evaluated and hand-searched.

The following search strategy was used: (prevalence OR seroprevalence OR frequency OR rate OR epidemiology) AND (“hepatitis C virus” OR “hepatitis C infection” OR “HCV” OR “viral hepatitis” OR hepatitis OR “hepatitis C antibodies”) AND (“injection drug users” OR “IDUs” OR “injection substance users” OR “injection drug use” OR “injection substance use” OR “intravenous drug users” OR “intravenous substance users” OR “drug users” OR “substance users” OR “drug injection” OR “substance injection” OR “drug addicts” OR “substance addicts” OR “injection drugs” OR “injection substances” OR “injecting drug” OR “injecting substance” OR “substance injection” OR “drug injection” OR “substance-injecting practice” OR “drug-injecting practice” OR “inject substance” OR “inject drug” OR “inject substance” OR “injecting drug users” OR “injecting substance users” OR “drug injection history” OR “substance injection history” OR “injection drug abusers” OR “injection substance abusers” OR “drug abusers” OR “substance abusers” OR “intravenous drug abuse” OR “intravenous substance abuse” OR “IV drug users” OR “IV substance users” OR “illicit drug injection” OR “illicit substance injection” OR “people who inject drugs” OR PWID OR “people who inject substances”) AND Iran. Differences in selected studies between two authors were resolved by consensus.

### Eligibility criteria

Eligible studies were identified according to the following PECOS (population, exposure, comparison/comparator, outcome and study type) criteria: i) population: Iranian population; ii) exposure: injection drug users; iii) comparison/comparator: type of substance injected and level of substance use, iv) outcome: HCV prevalence; and v) study type: cross-sectional study.

### Inclusion criteria

Inclusion criteria were as follows: i) studies were considered eligible if published in Persian or English; ii) published in peer-reviewed journals; iii) reporting HCV prevalence or with sufficient data, providing the possibility to calculate HCV prevalence among PWID; iii) using standardized tests to detect HCV, namely recombinant immunoblot assay (RIBA), polymerase-chain reaction (PCR) and enzyme-linked immunosorbent assay (ELISA); iv) devised as observational studies (of any kind: cross-sectional, cohort, or case-control); v) conducted in Iran; and vi) carried out without any limitations in terms of age and gender.

### Exclusion criteria

Exclusion criteria were as follows: i) studies were not deemed eligible if devised as case reports, case-series, reviews or systematic reviews (even though, if available, reference lists of reviews were scanned for ensuring a comprehensive coverage of the literature); ii) published as conferences abstracts or in proceedings; iii) not reporting HCV prevalence or not providing clear, suitable data for estimating HCV prevalence; iv) conducted among HIV-positive individuals or subjects with other disorders; v) not carried out in Iran; and vi) with overlapping/duplicate data.

### Screening and data extraction

After finding potentially related studies, authors extracted relevant data and information based on ad hoc Excel spreadsheet. Extracted data included the surname of the first author, the study journal, the year of publication, the number of participants examined, the type of diagnostic test performed, the number of positive HCV patients, the number of participants stratified by gender, the reported prevalence, the duration of drug injection practice and the history of using a shared syringe.

### Study quality appraisal

The Joanna Brigg’s Institute (JBI) checklist was used to check the quality of selected studies [20]. This checklist has 10 questions and is particularly suitable for the appraisal of epidemiological and prevalence studies. The answer to each question is yes, no, unclear or not applicable.

### Statistical analysis

For all data analysis, the commercial software STATA Ver.14 (Stata Corp, College Station, TX, USA) was used. Figures with *p*-values < 0.05 were considered statistically significant. To calculate HCV prevalence among Iranian PWID, a random-effect model according to the DerSimonian-Laird approach with the Freeman-Tukey double arcsine transformation with 95% confidence interval (CI) was used [21, 22]. For computing the amount of heterogeneity among studies, the I^2^ statistics was utilized [23]. The Egger’s linear regression test was used for assessing the presence of publication bias [24]. Based on the age of the participants, sample size, duration of injection (based on the selected studies, the mean duration of injection was calculated by the authors to be 3 years) and geographic region of the study, subgroup analyses were carried out. Furthermore, sensitivity analysis was performed in order to ensure the stability of the results. Also, to further investigate the possible sources of heterogeneity among studies, meta-regressions were conducted based on the year of study, sample size and age of participants.

## Results

**Fig. 1:**
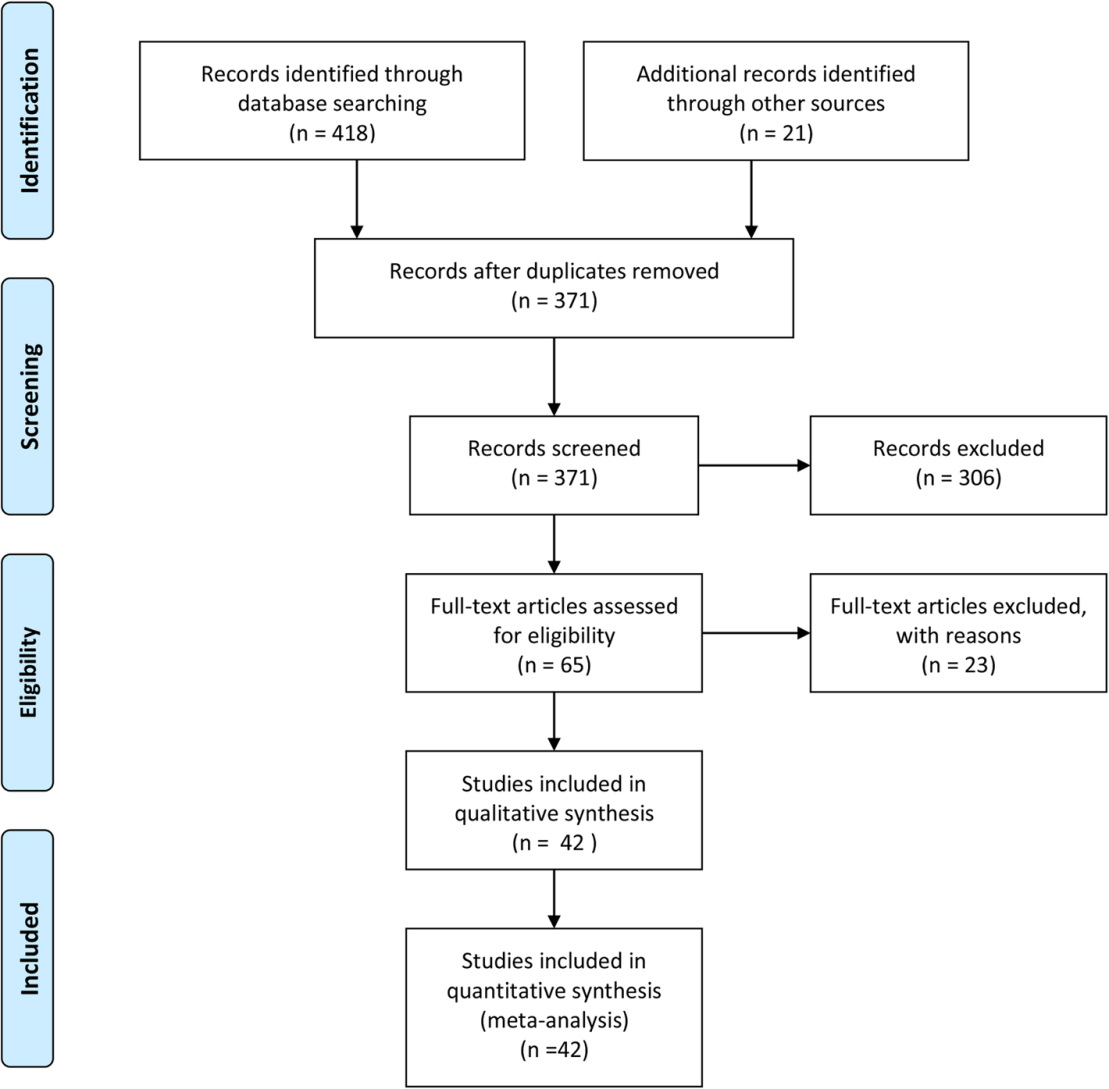
Process of searching, retrieving and selecting potentially relevant studies

The initial search of the literature yielded a pool of 474 records. Figure 1 shows the process of searching, retrieving and selecting relevant studies. 68 records were duplicate items and, as such, were removed. The title of the studies was then reviewed and at that step 321 records were removed. The abstract of the articles was reviewed and, finally, 42 studies were retained based on the above-mentioned inclusion and exclusion criteria [25–66].

Table 1 summarizes the characteristics of the 42 studies included in the present study (all 42 investigations were designed as cross-sectional studies). 15,072 PWID were assessed for determining the prevalence of HCV. Retained studies were published between 2001 and 2017. Mean age was ranging between 20 and 42 years. All studies used ELISA to detect HCV.

**Table 1:** Characteristics of the included studies

First name	Year	Mean age	Province	Sample	No-male	No-female	Test	Prevalence (%)
Zali	2001	34.2	Tehran	402	402	0	Elisa	45.3
Rowhani Rahbar	2004	NA	Khorasan Razavi	101	NA	NA	Elisa	59.4
Sarvghad	2005	NA	Khorasan Razavi	53	50	3	Elisa	67.92
Mohammad Alizadeh	2005	NA	Hamadan	149	NA	NA	Elisa	31.5
Imani	2006	33.4	Shahrekord	133	131	2	Elisa	11.2
Zamani	2007	30	Tehran	202	196	6	Elisa	52
Mohtasham Amiri	2007	34.7	Guilan	81	81	0	Elisa	88.9
Aminzadeh	2007	34.4	Tehran	70	NA	NA	Elisa	36
Imani	2008	33.4	Chaharmahal and Bakhtiari	133	131	2	Elisa	11.2
Mir-Nasseri	2008	36	Tehran	467	464	54	Elisa	NA
Soudbakhsh	2008	35.3	Tehran	60	60	0	Elisa	80
Kheirandish	2009	35	Tehran	454	NA	NA	Elisa	80
Mirahmadizadeh	2009	33	Tehran	1525	1465	60	Elisa	43.4
Sharif	2009	36.5	Isfahan	200	177	23	Elisa	10.5
Tajbakhsh	2009	NA	Shahrekord	90	NA	NA	Elisa	NA
Alavi	2009	26.3	Khozestan	142	NA	NA	Elisa	52.11
Davoodian	2009	35.4	Hormozgan	249	NA	NA	Elisa	64.8
Zamani	2010	23.6	Isfahan	117	114	3	Elisa	59.4
Merat	2010	NA	Tehran-Hormozgan-Golestan	40	NA	NA	Elisa	NA
Hosseini	2010	NA	Tehran	417	417	0	Elisa	80
Alavi	2010	24.8	Khozestan	333	323	10	Elisa	30.9
Mir-Nasseri	2011	NA	Tehran	392	464	54	Elisa	NA
Keramat	2011	NA	Hamadan	199	NA	NA	Elisa	NA
Kaffashian	2011	32.6	Isfahan	951	946	5	Elisa	42
Azizi	2011	NA	Kermanshah	58	NA	NA	Elisa	53.4
Mobasheri zadeh	2011	NA	Isfahan	1055	NA	NA	Elisa	7
Ataei	2011	NA	Isfahan	136	NA	NA	Elisa	19.8
Nokhodian	2012	16.59	Isfahan	6	NA	NA	Elisa	50
Nokhodian	2012	31.77	Isfahan	531	503	28	Elisa	47.1
Kassaian	2012	32.6	Isfahan	943	938	5	Elisa	41.6
Nobari	2012	35	Isfahan	1747	581	14	Elisa	34
Sofian	2012	30.7	Markazi	153	153	0	Elisa	59.5
Amin-Esmaeili	2012	33.9	Tehran	895	859	36	Elisa	NA
Tavanaei Sani	2012	NA	Khorasan Razavi	62	NA	NA	Elisa	71
Sarkari	2012	NA	Kohgiloyeh and Boyerahmad	158	NA	NA	Elisa	42.2
Honarvar	2013	NA	Fars	233	NA	NA	Elisa	40.3
Ziaee	2014	NA	Southern Khorasan	25	NA	NA	Elisa	NA
Ramezani	2014	33.3	Markazi	100	100	0	Elisa	56
Salehi	2015	20.24	Fars	222	NA	NA	Elisa	90
Afshari	2016	38.82	Fars	772	NA	NA	Elisa	14.2
Sharhani	2017	36.7	Kermanshah	606	NA	NA	Elisa	NA
Rezaie	2017	33.2	Kermanshah	410	410	0	Elisa	42

The critical methodological assessment of the quality of selected studies is presented in Table 2, showing the high methodological rigor of the included investigations.

**Table 2:** Methodological assessment of the quality of selected studies

Study	Was the sample frame appropriate to address the target population?	Were study participants sampled in an appropriate way?	Was the sample size adequate?	Were the study subjects and the setting described in detail?	Was the data analysis conducted with sufficient coverage of the identified sample?	Were valid methods used for the identification of the condition?	Was the condition measured in a standard, reliable way for all participants?	Was there appropriate statistical analysis?	Was the response rate adequate, and if not, was the low response rate managed appropriately?
Zali	Y	Y	Y	Y	Y	Y	Y	Y	Y
Rowhani Rahbar	Y	Y	Y	Y	Y	Y	Y	Y	Y
Sarvghad	Y	Y	N	Y	Y	Y	Y	Y	Y
Mohammad Alizadeh	Y	Y	Y	Y	Y	Y	Y	Y	Y
Imani	Y	Y	N	Y	Y	Y	Y	Y	Y
Zamani	Y	Y	U	Y	Y	Y	Y	Y	Y
Mohtasham Amiri	Y	Y	Y	Y	Y	Y	Y	Y	Y
Aminzadeh	Y	Y	Y	Y	Y	Y	Y	Y	Y
Imani	Y	Y	Y	Y	Y	Y	Y	Y	Y
Mir-Nasseri	Y	Y	Y	Y	Y	Y	Y	Y	Y
Soudbakhsh AR	Y	Y	Y	Y	Y	Y	N	Y	Y
Kheirandish	Y	Y	Y	Y	Y	Y	Y	Y	N
Mirahmadizadeh	Y	Y	N	Y	Y	Y	Y	Y	Y
Sharif	Y	Y	Y	Y	Y	Y	Y	Y	Y
Tajbakhsh	Y	Y	U	Y	Y	Y	Y	Y	Y
Alavi	Y	Y	Y	Y	Y	Y	U	Y	Y
Davoodian	Y	Y	Y	Y	Y	Y	Y	Y	Y
Zamani	Y	Y	Y	Y	Y	Y	Y	Y	N
Merat	Y	Y	Y	Y	Y	Y	Y	Y	Y
Hosseini	Y	Y	N	Y	Y	Y	Y	Y	Y
Alavi	Y	Y		Y	Y	Y	Y	Y	N
Mir-Nasseri	Y	Y	Y	Y	Y	Y	U	Y	Y
Keramat	Y	Y	Y	Y	Y	Y	Y	Y	Y
Kaffashian	Y	Y	Y	Y	Y	Y	Y	Y	Y
Azizi	Y	Y	N	Y	Y	Y	Y	Y	N
Mobasheri zadeh	Y	Y	Y	Y	Y	Y	Y	Y	Y
Ataei	Y	Y	N	Y	Y	Y	Y	Y	Y
Nokhodian	Y	Y	U	Y	Y	Y	Y	Y	Y
Nokhodian	Y	Y	N	Y	Y	Y	N	Y	Y
Kassaian	Y	Y	Y	Y	Y	Y	N	Y	N
Nobari	Y	Y	Y	Y	Y	Y	Y	Y	Y
Sofian	Y	Y	Y	Y	Y	Y	Y	Y	Y
Amin-Esmaeili	Y	Y	Y	Y	Y	Y	Y	Y	Y
Tavanaei Sani	Y	Y	Y	Y	Y	Y	Y	Y	Y
Sarkari	Y	Y	Y	Y	Y	Y	Y	Y	U
Honarvar	Y	Y	U	Y	Y	Y	Y	Y	
Ziaee	Y	Y	Y	Y	Y	Y	Y	Y	U
Ramezani	Y	Y	N	Y	Y	Y	Y	Y	Y
Salehi	Y	Y	Y	Y	Y	Y	Y	Y	Y
Afshari	Y	Y	Y	Y	Y	Y	Y	Y	Y
Sharhani	Y	Y	Y	Y	Y	Y	Y	Y	Y
Rezaie	Y	Y	Y	Y	Y	Y	Y	Y	Y

(*Y* Yes, *N* No, *U* Unclear, *NA* Not Applicable)

**Fig. 2:**
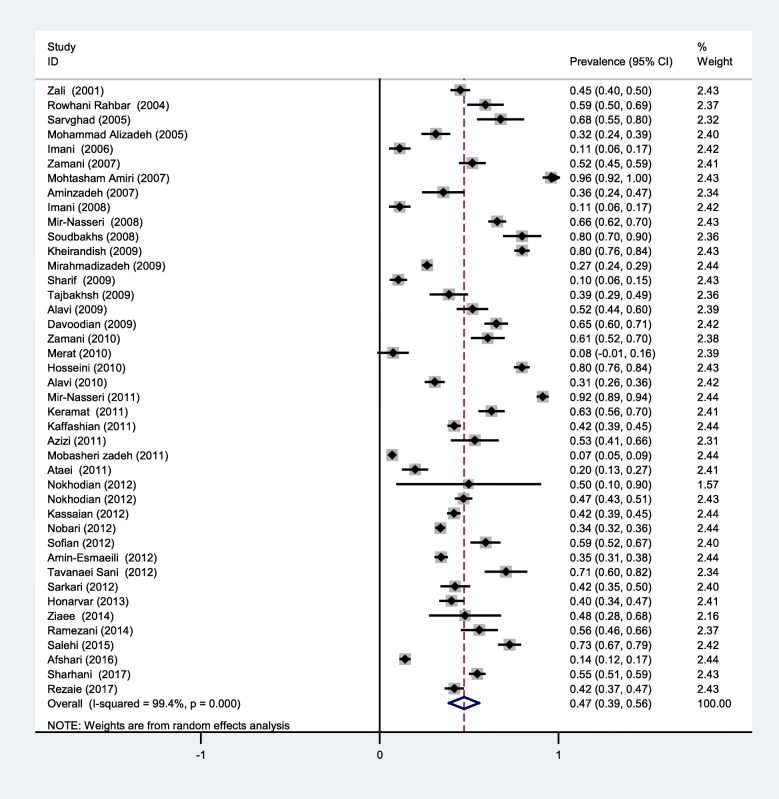
The forest plot of the studies included in the present systematic review and meta-analysis

According to the DerSimonian-Laird random-effect model, the overall prevalence of HCV among PWID in Iran was computed to be 47% (CI 95: 39–56). The prevalence ranged between 7 and 96%. The I^2^ statistics yielded a value of 99.4%, indicating high, statistically significant heterogeneity among studies. Figure 2 shows the forest plot of the selected studies.

Several subgroup-analyzes were conducted to explore the sources of heterogeneity among studies. Table 3 shows the results based on sample size, imprisoned PWID, geographic regions, age, and duration of injection.

**Table 3:** Sub-group analyses based on sample size, geographical region, age, duration of injection and type of study

Variable	No study	Prevalence 95% Confidence interval (CI)	I2	*P* – value
Sample size				
≤ 200	22	56% (44–68)	99%	0.00
> 200	20	37% (29–46)	98.1%	0.00
Region				
North	1	26% (22–30)	–	–
West	9	39% (26–53)	98.6%	0.00
South	9	59% (41–78)	96.8%	0.00
East	4	61% (43–76)	92.2%	0.00
Central	19	44% (31–62)	97.3%	0.00
Age				0.00
≤ 30	7	53% (35–71)	94.3%	0.00
> 30	20	45% (33–56)	98.4%	0.00
NA	15	49% (33–64)	97.3%	0.00
Duration of injection (years)				
≤ 3	12	57% (26–68)	92.7%	0.00
> 3	11	52% (41–83)	98.3%	0.00
NA	19	48% (34–59)	97.1%	0.00
Type of participants				
Only prisoners	10	52% (38–67)	98.5%	0.00
Non-prisoners	28	45% (36–54)	99.2%	0.00
Both (Non-prisoners and prisoners)	4	53% (17–89)	99.6%	0.00

The sensitivity analysis was performed and based on it the results before and after removing each study per time did not change, showing that the results were stable and reliable.

**Fig. 3:**
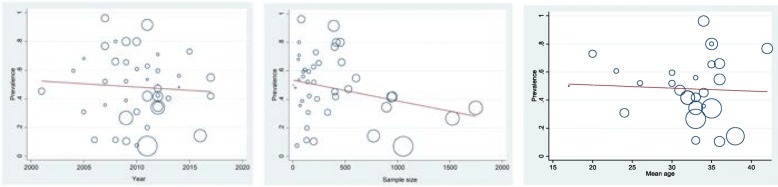
Findings of the meta-regressions based on sample size, year of publication and mean age of participants

Based on sample size, year of publication and mean age of participants, meta-regression analyses were conducted. The findings showed that the prevalence tended to decrease by sample size (*P* = 0.063) and year of publication (*P* = 0.039), both statistically significant. Similarly, as the age increased, the prevalence declined in a statistically significant fashion (*P* = 0.061). Figure 3 shows the results of the meta-regressions.

**Fig. 4:**
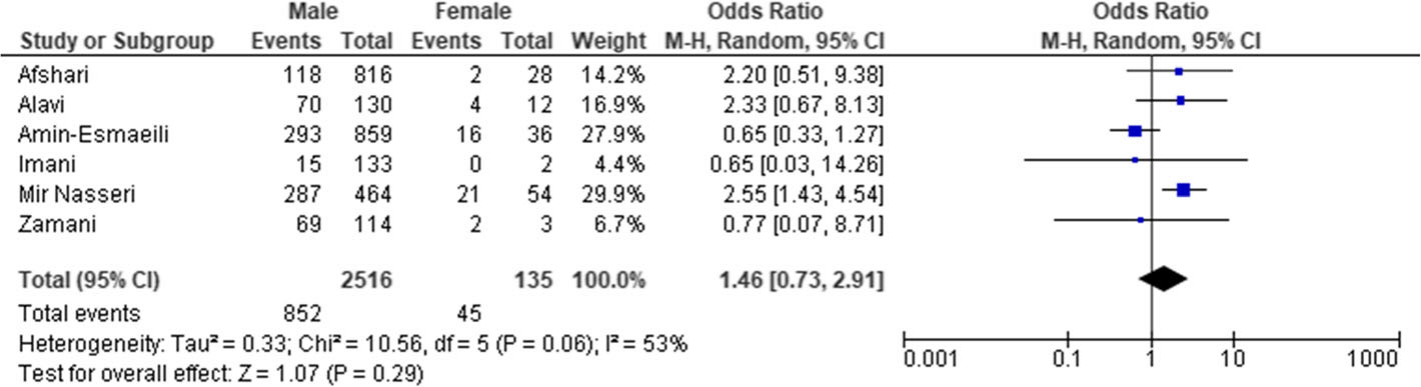
HCV prevalence stratified by gender

Six studies provided data that enabled to estimate the odds ratio (OR) of HCV infection in terms of gender. The OR for HCV among PWID men was about 1.46 times that of women, statistically significant and suggesting that men were at higher risk of developing HCV compared to women (Fig. 4).

**Fig. 5:**
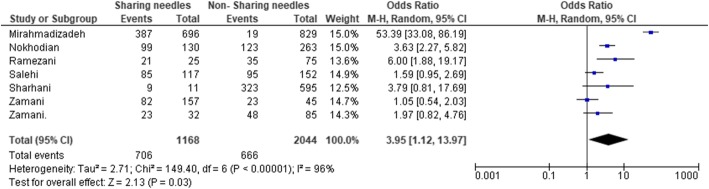
HCV prevalence stratified by the history of sharing needles

Some studies assessed the impact of using a common/shared syringe showing that individuals who used a common syringe for injection were 3.95 times more likely to be at risk. Figure 5 shows the odds ratio for using a common syringe.

**Fig. 6:**
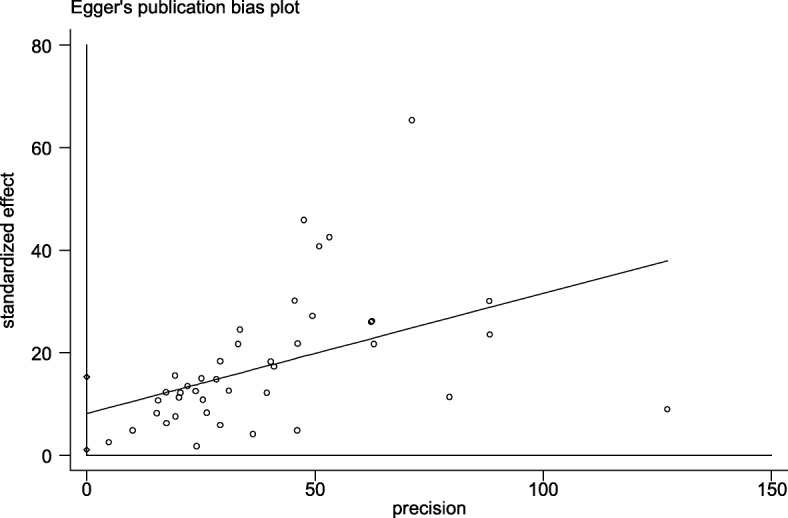
Egger’s linear regression test, showing no presence of publication bias

Publication bias was evaluated by performing the Egger’s linear regression test and we could not find any evidence of publication bias in included studies according to *P* = 0.23 (Fig. 6).

## Discussion

Planning to implement appropriate and effective programs to prevent and control diseases requires the use of robust and updated scientific evidence. The aim of this study was to determine the prevalence of HCV among the PWID, a well-known high-risk group for developing HCV. The findings of this study showed that the prevalence was 47%. This was higher than the prevalence of HCV among prisoners (28%) [67], thalassemia patients (19%) [68], street children (2.4%) [69], blood donors (0.5%) [70] and the general population (0.6%) [14]. This high rate confirms that, as it is well-known, PWID are one of the most important and high-risk groups for developing HCV [2, 71].

The prevalence of HCV among PWID in Iran was lower than the rate reported in other countries, including South Africa (55%) [72], Pakistan (72%) [73] and India (53.7%) [74] but higher than in studies conducted in Kuwait (12.28%) [4], Kingdom of Saudi Arabia (42.7%) [75], and Brazil (35.6%) [76]. These differences in prevalence can be attributed to differences in health systems, screening methods, and the type of high-risk behaviors of individuals [77, 78]. In particular, in developing countries, harm reduction programs such as syringe distribution are not fully implemented [79]. Furthermore, because of the high cost of diagnostic services, many PWID are not aware of their health status. The high cost of management and the lack of referral for treatment can also be some of the reasons explaining the contrasting findings among various studies [80, 81].

The findings of this study showed that the highest prevalence of HCV among PWID in Iran was observed in East and South of Iran (60%). Neighborhood of countries such as Afghanistan and Pakistan could explain, at least partially, such findings [82]. Moreover, there are a lot of harbors in Southern Iran, which is one of the ways to transport narcotic drugs to other countries. For this reason, addicts in the area from Iran have easy access conditions. One of the most important policies implemented by the government in Iran is a serious struggle against the narcotics commerce and sale, with the support of international organizations [83].

Moreover, the findings of this study showed that the prevalence of HCV in men was higher than that among women, which was consistent with the results of studies performed in India [84] as well as in upper middle-income countries [78]. Men were found to be more likely to be at risk than women, probably due to differences in lifestyles and behaviors that make men more susceptible to HCV. The cultural and social conditions in Iran have led men to become more oriented toward injecting drug use than women. As such, most of the participants evaluated in the studies included in the present systematic review and meta-analysis were male.

The findings showed that the prevalence of infections in people who had a history less than 3 years of injection was higher than the rate among those who had an injection history longer than 3 years. A reason could be the early detection of the disease in these people. Unfortunately, one of the problems with HCV is that many people are not aware of their illness, not having access to diagnostic services, due to the expensive testing costs, and the lack of motivation to diagnose possible illnesses [27, 52, 58, 65].

PWID that had a history of sharing needles had a 3.95-fold increased risk of developing HCV infection, which is in line with the literature [4, 85]. Various studies have shown that needle exchange programs (NEP) can be used as a harm reduction policy to decrease HCV prevalence among PWID, even though the effectiveness of this program has yet to be properly verified [86, 87].

Meta-regressions showed that the prevalence of HCV among PWID in Iran has decreased in recent years, even though not in a statistically significant way. This decrease reflects the widespread effort to reduce HCV-related risk of diffusion and transmission. Health policy- and decision-makers in Iran have adopted valuable harm reduction policies to prevent and control HCV among high-risk groups, and especially PWID. The Ministry of Health, as the most important actor in controlling the disease, has been developing educational programs for the general population, as well as for the high-risk groups. Establishing centers in all provinces for the distribution of syringes, condoms and alternative treatments such as methadone has enabled to obtain good results. In these centers, diagnostic tests are performed free of charge for PWID and others who have high-risk behaviors. The support of the judiciary system in Iran has led to a serious emphasis on screening programs in Iranian prisons. There are also special centers for women who offer harm reduction services. All these activities have contributed to controlling the disease.

The findings of the present study showed that the prevalence of HCV was higher in studies involving only prisoners (52%) compared to studies involving non-prisoners (45%). Prison for individuals can be an important risk factor for injecting drug use (IDU) [88]. The absence or inappropriate implementation of risk reduction policies in prisons around the world has led to serious problems for prisoners [89]. The pattern of drug use in Iran has changed in recent decades and IDUs are on the rise [67]. In one sense, prisoners practicing IDU are more susceptible to infections such as HBV, HCV and HIV than others [90].

However, this study has some limitations, which should be properly acknowledged. Epidemiological studies of HCV prevalence among PWID were not performed in all provinces and areas of Iran. Therefore, it is necessary to conduct observational surveys in all provinces in order to obtain a clear understanding of the disease condition. High amounts of heterogeneity as shown by the I^2^ statistics indicate that there are methodological differences among studies, as indicated also by the subgroup analysis. Other limitations include the lack of sufficient quantitative data from some studies that hindered the possibility of computing HCV prevalence, especially stratifying by age-groups and gender.

## Conclusion

The findings of the present study showed that the prevalence of HCV among PWIDs in Iran is high. The support and implementation of ad hoc health-related policies and programs that reduce this should be put into action. Alternative treatments such as methadone therapy and HCV therapy can help control the problem. Health policy- and decision-makers in Iran should provide faster diagnosis and access to low-cost health care.

## Data Availability

Not applicable.

## Abbreviations

DOAJ: Directory of Open Access Journals
ELISA: Enzyme-linked immunosorbent assay
HCV: Hepatitis C virus
IDU: Injecting drug use
JBI: Joanna Brigg’s Institute
OR: Odds ratio
PCR: Polymerase-chain reaction
PRISMA: Preferred Reporting Items for Systematic Reviews and Meta-Analyses
PWID: People who inject drugs
RIBA: Recombinant immunoblot assay
WHO: World Health Organization
